# Sleep quality and its psychological correlates among university students in Ethiopia: a cross-sectional study

**DOI:** 10.1186/1471-244X-12-237

**Published:** 2012-12-28

**Authors:** Seblewngel Lemma, Bizu Gelaye, Yemane Berhane, Alemayehu Worku, Michelle A Williams

**Affiliations:** 1 Addis Continental Institute of Public Health, Addis Ababa, Ethiopia; 2 Department of Epidemiology, Harvard School of Public Health, Boston, MA, USA; 3 School of Public Health, Addis Ababa University, Addis Ababa, Ethiopia

**Keywords:** Pittsburgh sleep quality index, Perceived stress, Anxiety, Depression, Students, Ethiopia

## Abstract

**Background:**

Sleep is an important physiological process for humans. University students in most resource limited countries often report poor sleep quality due to changing social opportunities and increasing academic demands. However, sleep quality among university students has not been studied in Ethiopia. Thus, this study assessed sleep quality and its demographic and psychological correlates among university students.

**Methods:**

A cross-sectional survey was conducted in two universities in Ethiopia. Multistage sampling procedures were used to enroll 2,817 students into the study. A self-administered structured questionnaire including the Pittsburgh Sleep Quality Index (PSQI), the Depression Anxiety Stress Scale-21, the Perceived Stress Scale (PSS) and selected modules of the World Health Organization STEPS instrument was used for the study. This research included 2,551 students. Frequency, median, mean with standard deviation and 95% confidence interval were used to characterize sleep quality and other variables. Analysis of variance and binary logistic regression procedures were also used.

**Result:**

The prevalence of poor sleep quality (total PSQI score > 5) was 55.8% (1,424). Female students (adjusted odds ratio (AOR) 1.23; 95% CI: 1.00, 1.57), second year (AOR 2.91; 95% CI: 2.1, 4.02) and third year students (AOR 2.25; 95% CI 1.62, 3.12) had statistically significant higher odds of poor sleep quality. Perceived stress level and symptoms of depression and anxiety were strongly associated with sleep quality.

**Conclusion:**

A substantial proportion of university students are affected by poor sleep quality. If our results are confirmed in prospective studies, health promotion and educational programs for students should emphasize the importance of sleep and mental health.

## Background

Sleep is an important physiological process for humans. Although the direct benefits of sleep is not well quantified across many populations, it is understood that sleep deprivation has serious health consequences [1]. The quality of sleep is a measure of both the quantitative and qualitative components of sleep. The quantitative component includes the duration of sleep while the qualitative component is a subjective measure of the depth and feeling of restfulness upon awakening [2].

Reductions in sleep duration and sleep quality, across populations, has been linked to changes in lifestyle, increasing use of technology and increased work and social demands [3]. Of note, investigators have identified university students as particularly susceptible to these increasing demands [4]. This thesis is underscored, by the fact that the transition from secondary school to university is characterized by reduced adult supervision, new social opportunities with its commitments, difficult studies and other extracurricular activities resulting in irregular sleep schedules and higher risks for sleep deprivation [3,5]. Moreover, investigators have reported high prevalence estimates (≥40%) of short sleep duration (< 7 hours) [5-7] and poor sleep quality as measured by Pittsburgh Sleep Quality Index (PSQI) [8-10] among university students. Short sleep duration has also been documented in the few studies that have focused on university students in sub-Saharan African countries [11,12].

Sleep quality and duration are generally known to vary by sex and age, though findings are in consistent across studies [5,7,12]. In some studies female students have been identified as having a higher risk of poor sleep quality [13-15]. However, some studies suggest that female students have longer mean sleep durations [12]. Increased age is also associated with indices of poor sleep quality [16], in some but not all studies [7,13]. Inconsistencies may be attributable with the narrow age range of university students included in some studies.

Psychological correlates such as stress, anxiety and depressive symptoms are commonly reported phenomena among university students [17-20]. The sources of such psychiatric morbidities are reported to be academic workload and psychosocial concerns [7,17,18,20,21]. Evidence from both cross-sectional and longitudinal studies have also documented associations of different sleep indices with symptoms of depression [19,22-25], stress [7,26] and anxiety [22-24] among university students. Given the high prevalence of sleep and mental health problems among young adults, particularly among university students; and given the absence of published reports that have simultaneously evaluated the prevalence and comorbid relationships of sleep and mental health problems among Ethiopian students, we developed the present study. We expect that findings from this study will help to identify factors influencing poor sleep. We also expect that our study will provide objective evidence that may be used to guide the development of health and wellness programs for young adults in Ethiopia and other East African settings.

## Methods

### Study design and setting

A cross sectional study was conducted in two universities (Haramaya University, in Eastern Ethiopia and University of Gondar in North West Ethiopia) in Ethiopia. Study participants were undergraduate regular students from second year to final year. First year students were excluded from the study because they were not yet admitted or fully registered at the start of the present study.

### Sample size and sampling procedure

A multistage sampling design by means of probability proportional to size (PPS) was used to select departments for participation in the research. This approach was performed for both universities, and all students in selected departments were invited to participate. Students who expressed an interest in participating in the study were invited to meet in their classroom where they were informed about the purpose of the study and asked to participate in the survey. A random sample of these students was then selected and asked to complete a self-administered individual survey after providing consent. There was no set time limit for completing the survey. Students who could not read the survey (i.e., were blind) were excluded, as were those enrolled in correspondence, extension, or night school programs. A total of 2,817 undergraduate students participated in the study, the participation rate was 94%. After excluding students with incomplete questionnaires and missing sleep quality scores, the final analyzed sample consisted of 2,551 students. Based on the information provided, students excluded from analysis had similar characteristics to those considered. All completed questionnaires were anonymous, and no personal identifiers were used.

### Data collection instruments and variables

A bi-lingual self-administered questionnaire was used to collect information for this study. The questionnaire was initially developed in English and was then translated by Ethiopian linguist experts into Amharic, the lingua franca for the country. The final bi-lingual questionnaire was then evaluated by experts fluent in both Amharic and English. Finally, the questionnaire was pilot tested in students at other universities in Ethiopia before being deployed at the participating universities. The questionnaire ascertained demographic information including age, sex, and education level. Behavioral factors such as alcohol consumption were assessed using the WHO STEPS instrument [27] while khat use (a stimulant plant widely used in East Africa and Arabian Peninsula) [28] was measured adopting a set of questions.

Sleep quality was assessed using the previously validated Pittsburgh Sleep Quality Index (PSQI) [2]. The PSQI consists of 24 questions which generate seven component scores each ranging from 0 to 3 (0 score equals better and 3 is worst) and one global sleep quality score. The global score > 5 designates poor sleep quality while score ≤ 5 is considered good quality sleep [2]. The instrument has been validated in different population including university students [2,7,11].

Depression, anxiety, and stress symptoms were measured using the Depression Anxiety and Stress Scale (DASS 21) which consists 21 questions; seven under each of the three negative affective states. The questions inquire the experience of the items in the past week and each item is scored from 0 (did not apply to me at all) to 3 (applied to me very much). Finally the values obtained were multiplied by 2. The reliability of these instruments was checked using the Cronbach α and it was 0.82 for depression, 0.78 for anxiety and 0.79 for stress components. Table 1 depicts previously suggested cut of values for each component of DASS-21 score [29]. Categorization of study participants according to symptom levels of stress, depression and anxiety were made using cut off points suggested in the literature [29]. See Table 1.

**Table 1:** Depression, anxiety and stress score severity ratings

	**Depression**	**Anxiety**	**Stress**
Normal	0-9	0-7	0-14
Mild	10-13	8-9	15-18
Moderate	14-20	10-14	19-25
Severe	21-27	15-19	26-33
Extremely Severe	28+	20+	34

The cut of values are established based on previous literature [29].

The Perceived Stress Scale (PSS) was also used to measure the perception of stress. The PSS is a 10 item instrument that include questions about ones feelings and thoughts in the last one month (how often one felt in a certain way) with a 5 point Likert scale (0 = never, and 4 = very often). The score ranges from 0 to 40 and higher score represented higher level of perceived stress [30]. The psychometric property of this instrument was also studied in other studies and favorable results were documented [31,32]. In this study, reliability of the instrument was also measured using Cronbach α and it was 0.76.

### Statistical analysis

Frequency, median, mean with standard deviation and 95% confidence intervals were used to characterize sleep quality and duration characteristics and other demographic and behavioral characteristics. Analysis of variance (ANOVA) procedures were used to assess mean differences in sleep quality, stress, and depression and anxiety scores according to number of years at university. A post-hoc analysis using Bonferroni correction methods was made for those with a statistically significant difference.

Multi-level analysis techniques were used to measure associations of demographic and psycho-social factors with the measures of sleep quality [33]. The group level variable considered was year of stay in the university. Fitting the selected variable on the multilevel mixed model, the intra cluster correlation coefficient was calculated and only 4.5% of the variance in the PSQI score was attributed to the group level variation. Thus, binary logistic regression was used to evaluate the association between the set of demographic and psychological factors and poor quality sleep. The adjusted odds ratio with the 95% CI and the P-values are reported.

### Ethical consideration

All completed questionnaires were anonymous, and no personal identifiers were used. Approval to conduct this study was obtained from the deans of all participating universities. The procedures used in this study were approved by the Addis Continental Institute of Public Health (ACIPH) and University of Gondar Institutional Review Board (IRBs), also from University of Washington Human Subject Committee. Permission was also obtained from Gondar and Haramaya Universities. Informed consent was obtained from each participant.

## Results

### Demographic and psychological factors

The response rate in this study was 94%. After excluding participants with missing information for sleep quality, a total of 2,551 students remained for analysis. More than fifty percent (1,298) of students were from Haramaya University and more than seventy five percent (1,940) were males. The median age was 21 years [range 17 to 35 years]; and 88.6% (2,465) of students were between 20 and 24 years of age. Most students were sophomores and juniors 49.4% (1,258) or 42.2% (1,074) respectively. See Table 2.

**Table 2:** Demographic characteristics and psychological morbidities among university students

**Variables (N = 2551)**		**Frequency**	**Percentage**
Sex	Female	574	22.8%
	Male	1940	77.2%
Age	15-19	134	5.3%
	20-24	2243	88.6%
	25 +	156	6.2%
Perceived Stress Scale	1^st^ quartile	747	29.3%
Mean Score (SD) = 16.3(5.9)	2^nd^ quartile	587	23.0%
	3^rd^ quartile	649	25.4%
	4^th^ quartile	568	22.3%
Depression	Normal	1257	49.3%
Mean score (SD) = 10.6(8.6)	Mild	392	15.4%
	Moderate	583	22.9%
	Severe	200	7.8%
	Extremely severe	119	4.7%
Anxiety	Normal	1182	42.0%
Mean score (SD) = 10.1(8.1)	Mild	271	9.6%
	Moderate	628	22.3%
	Severe	319	11.3%
	Extremely severe	417	14.8%
Stress	Normal	1687	66.1%
Mean Score (SD) = 12.4(8.0)	Mild	400	15.7%
	Moderate	297	11.6%
	Severe	130	5.1%
	Extremely severe	37	1.5%

**Figure 1:**
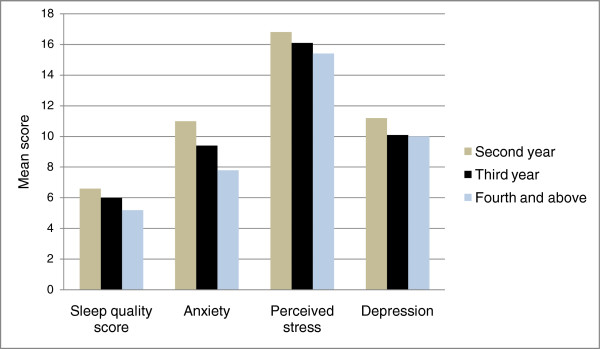
The mean scores of sleep quality and the psychological correlates distribution by year of study in the university.

Approximately 50.8% (1,294) of the students reported mild to extremely severe depressive symptoms, 58% (1,369) of students reported mild to extremely severe levels of anxiety symptoms, and 34.1% (864) of students reported mild to extremely severe levels of stress seven days prior to the study. Using the perceived stress scale score, 48% (1,217) were in the third and fourth quartile of the scale four weeks prior to the survey (Table 2). Mean scores of perceived stress, anxiety and depression were statistically significantly reduced with increasing numbers of years in university (P-values <0.005 for both stress and anxiety; P-value = 0.005 for depression) (Figure 1). According to the post hoc analysis, significant mean differences in depression scores were accounted for by differences between second and third year students with the P-value of 0.006. For perceived stress, the differences were between second and third year, second year and fourth year and third year and fourth year (p<0.001). For anxiety, the significant difference between second year and third year; second year and fourth year were important with the following p-values 0.008 and 0.004 respectively.

### Sleep quality and its component scores

The mean global PSQI score (computed using the component scores) was 6.23, 95% CI (6.12, 6.35). Overall, 55.8% (1,424) of students were classified as having poor sleep quality. The mean duration of sleep per night was reported to be 6.79 ± 1.9 hours with 95% CI (6.7, 6.8). Of note, and only 37.6% (960) reported sleeping > 7 hours. Mean PSQI scores were statistically significantly associated with years of study at university (P <0.005) (Figure 1); the differences were between second and third year, second year and fourth year and third year and fourth year (p<0.001). The median amount of sleep latency was 30 minutes with inter-quartile range of 20 minutes. Of note, when assessment was limited to the component item that measured self-report of poor sleep quality, only 12.4% (314) of the students specifically stated that their sleep quality was fairly bad or very bad. Self-reports of day time dysfunction was less frequent, with 6.1% (155) of students in the highest difficulty category while the habitual sleep efficiency of less than or equal to 74% was reported by 19.4% (418) of the participants. The second highest sleep disturbance score was also reported by 26.9% (686) of the participants and use of medication in the last one month was reported by 8.7% (245) of the participants. Table 3.

**Table 3:** Sleep quality and its components scores among university students

**Variables (N = 2551)**		**Frequency**	**Percentage**
Sleep Duration	Greater than 7 hrs	960	37.6%
Mean (SD) = 6.79(1.84)	6-7 hrs	1060	41.6%
	5-6 hrs	286	11.2%
	Less than 5 hrs	245	9.6%
Sleep latency	0	478	18.7%
	1	859	33.7%
	2	918	36.0%
	3	296	11.6%
Day time dysfunction	0	519	20.3%
	1	1267	49.7%
	2	610	23.9%
	3	155	6.1%
Sleep efficiency	>85%	1790	70.2%
	75-84%	343	13.4
	65-74%	173	6.8%
	<65%	245	9.6%
Subjective Sleep quality	Very good	852	33.4%
	Fairly good	1385	54.3%
	Fairly bad	236	9.3%
	Very bad	78	3.1%
Sleep disturbance	0	125	4.9%
	1	1722	67.4%
	2	686	26.9%
	3	18	0.7%
Use of sleep medication	Not during the past month	2326	91.2%
	Lessa than once a week	136	5.3%
	Once or twice a week	65	2.5%
	Three or more times a week	24	0.9%
Sleep quality score	Good sleep	1127	44.2%
Mean (SD) = 6.23(2.89)	Poor sleep	1424	55.8%

### Factors associated with sleep quality

Binary logistic regression was used to assess factors associated with sleep quality. Three multivariable models were used to explore these factors. The first model considered the demographic variables such as sex, age and other miscellaneous variables as years of study and university. The second model considered psychological factors such as perceived stress level, as well as anxiety and depression. The last model included both demographic and psychological factors as well as behavioral factors such as coffee consumption, cigarette, alcohol and khat use.

Female sex was not significantly associated with poor sleep quality in the first model but in the last model with AOR = 1.23 95% CI: (1.00, 1.57). Compared to students in 4^th^ year or above, second year [AOR = 2.91 95% CI: (2.10, 4.02)] and third year students [AOR = 2.25, 95% CI: (1.62, 3.12)] had higher odds of poor sleep quality. The odd of poor sleep quality has shown reduction as the students year of study increase; besides, the magnitude of effect reduced slightly for second year students and increased for third years in the final model. Students from Gondar University had 36% higher odds of poor sleep quality than Haremaya University students [AOR = 1.36, 95% CI: (1.13, 1.63)]. The magnitude of effect also showed increment in the last model.

The level of perceived stress scale has shown significantly increasing odds of poor sleep quality across the quartiles with the following odds ratio (95% CI) [1.35 (1.10, 1.70)], [1.55 (1.23, 1.96)] and 1.90 (1.46, 2.46)] respectively. Similarly, for depression, the odds ratio increased as the level of depression increased from mild to extremely severe with the following odds ratio (95% CI) [1.36 (1.06, 1.75)], [1.64 (1.27, 2.11)], [1.64 (1.11, 2.42)], [2.65 (1.56, 4.49)]respectively; the magnitude of effect has shown slight increment in the final model. Lastly, for anxiety, moderate to extremely severe levels of anxiety were significantly associated with poor sleep quality compared to the normal level with [AOR = 1.27 95% CI: (1.002, 1.61)] for moderate, [AOR 1.62 95% CI: (1.17, 2.24)] for severe and [AOR = 1.76 95% CI: (1.27, 2.45)] for extremely severe levels of anxiety. The magnitude of effect was attenuated in the final model for all categories. See Table 4.

**Table 4:** Demographic and psychological correlates of sleep quality among university students

**Characteristics**		**Model-I (N = 2511) Adj. OR (95% CI)**	**Model-II (N = 2551) Adj. OR (95% CI)**	**Model-III (N = 2511) Adj. OR (95% CI)**
Sex	Male	1		1
	Female	1.14 (0.94, 1.38)		1.23 (1.00, 1.57)*
Year of Study	2^nd^ year	2.97 (2.18, 4.04)**		2.91 (2.1, 4.02)**
	3^rd^ year	2.14 (1.57, 2.92)**		2.25 (1.62, 3.12)**
	≥4^th^ year	1		1
University	Haramaya	1		1
	Gondar	1.22 (1.04,1.44)*		1.36 (1.13, 1.63)*
Perceived stress	1st quartile		1	1
	2^nd^ quartile		1.42 (1.13, 1.77)*	1.35 (1.10, 1.70)*
	3^rd^ quartile		1.66 (1.32, 2.08)**	1.55 (1.23, 1.96)**
	4^th^ quartile		2.11 (1.64, 2.72)**	1.90 (1.46, 2.46)**
Depression	Normal		1	1
	Mild		1.26 (0.99, 1.61)	1.36 (1.06, 1.75)*
	Moderate		1.48 (1.16, 1.90)*	1.64 (1.27, 2.11)**
	Severe		1.62 (1.11, 2.37)*	1.64 (1.11, 2.42)*
	Extremely severe		2.38 (1.42, 4.00)*	2.65 (1.56, 4.49)**
Anxiety	Normal		1	1
	Mild		1.33 (1.00, 1.77)*	1.26 (0.94, 1.70)
	Moderate		1.31 (1.04, 1.66)*	1.27 (1.00, 1.61)*
	Severe		1.82 (1.32, 2.49)**	1.62 (1.17, 2.24)*
	Extremely severe		1.94 (1.41, 2.67)**	1.76 (1.27, 2.45)*

*p-value <0.05, **p-value < 0.01.

N.B. The third model is controlled for coffee, khat, alcohol, and cigarette consumption. Age was excluded from the model as it has strong correlation with year of study in University.

## Discussion

A significant proportion (55.8%) of students was classified as having poor sleep quality. After controlling for important demographic, behavioral and psychological factors, females, second year and third year students had significantly higher odds of poor sleep quality. Perceived stress level and symptoms of depression were strongly associated with poor sleep quality. Additionally, moderate to extremely severe levels of anxiety were related with poor sleep quality.

The proportion of students with poor sleep quality in our study population generally consistent with reports from previous reports [7,8,10,14]. The significant sex difference in sleep quality was also consistent with other studies conducted among students [9,13-15]. This difference by sex could be explained by the significantly higher proportion of female students reported shorter sleep duration and bad subjective sleep quality in this study. Other studies have also reported that female students were more likely to report longer sleep latency [15,34], sleep disturbance [5] and lower rating of their sleep quality [15].

We found that increasing year of study in university was associated with reduced odds of poor sleep quality. This observation is inconsistent with reports from the study of university students in Hong Kong [13]. Another study found higher proportion of short sleep duration among freshman students and longer sleep latency among seniors; however the second study did not find any difference on overall sleep quality by year of study in university [15]. Variations in results across studies may be explained by differences in social, academic demands across universities. Within our own study population, sleep quality varied according to university, where students from the University of Gondar had higher odds of poor sleep quality than other students.

Consistent with previous studies [19,22,23,26], we noted that student mental health status variables were associated with poor sleep quality. For example, perceived stress level and depression were strongly associated with sleep quality. The consistency of our findings with those of the published literature underscore the public health importance and implications of more thoroughly investigating links between sleep habits and sleep problems with mental health and wellness among young adults.

The findings of our study should be interpreted in light of some study limitations. First, given the cross-sectional nature of our study, it is difficult to determine whether poor sleep quality is a result of mood, anxiety and stress symptoms, or whether these psychological symptoms contributed to poor sleep. Second, our use of a self-administered survey that relied on subjective measures of sleep quality and other covariates may have introduced some degree of error in reporting behavioral covariates. However, we believe that these issues are in part reduced by our use of anonymous questionnaire and validated instruments. Furthermore, a thorough pre-testing of our questionnaire likely reduced the possible risk of ambiguity of the questions. Finally, non-response by approximately 9% of enrolled students, may have contributed to some selection bias in our study. However, our evaluation of available data on responders and non-responders suggest similarity across the two populations, thus reducing the level of concern about selection bias.

## Conclusions

In conclusion, poor sleep quality is highly prevalent among Ethiopian university students and significant associations with measures of mental health status exists. Our study confirms and expands the literature by focusing on young adults. University students in Ethiopia, and possibly other parts of East Africa, should be made aware of the observed sleep problems and comorbid mental health outcomes. Improved sleep quality will likely benefit university students in their mental health status, daily activities and academic performance. Educational campaigns focused on helping university avoiding the build-up of a chronic sleep debt may be important in enhancing the academic performance and in reducing the development of psychiatric disorders later in life.

By so doing, Ethiopia may be better positioned to reap the benefits of their efforts of expanding access to higher education to Ethiopian youths and young adults.

## Abbreviations

ACIPH: Addis continental institute of public health; ANOVA: Analysis of variance; AOR: Adjusted odds ratio; CI: Confidence interval; DASS-21: Depression anxiety stress scale-21; IRB: Institutional review board; PPS: Proportional to population size; PSQI: Pittsburgh sleep quality index; PSS: Perceived stress scale (PSS); WHO: World health organization.

## Competing interests

The authors declare that they have no competing interests.

## Authors’ contributions

SL was involved in planning the study, organized data collection, performed analyses and drafted the manuscript. YB and AW planned the study and supervised analyses and drafted the manuscript. MAW and BG planned the study and drafted the manuscript. All authors read and approved the final manuscript.

## Pre-publication history

The pre-publication history for this paper can be accessed here:

http://www.biomedcentral.com/1471-244X/12/237/prepub
